# Toxicological Evaluation of Novel Cyclohexenone Derivative in an Animal Model through Histopathological and Biochemical Techniques

**DOI:** 10.3390/toxics9060119

**Published:** 2021-05-25

**Authors:** Muhammad Kamil, Arifa Fatima, Sami Ullah, Gowhar Ali, Rasool Khan, Naila Ismail, Mughal Qayum, Marius Irimie, Catalina Georgeta Dinu, Hanadi Talal Ahmedah, Maria Elena Cocuz

**Affiliations:** 1 Department of Pharmacy, University of Peshawar, Peshawar 25120, Pakistan; muhammadkamil_32@yahoo.com (M.K.); exoneratedsoul91@gmail.com (A.F.); gowhar_ali@uop.edu.pk (G.A.); 2 Department of Organic Chemistry, Institute of Chemical Sciences, University of Peshawar, Peshawar 25120, Pakistan; rasoolkhan@uop.edu.pk; 3 Department of Pathology, Kabir Medical College, Gandhara University, Peshawar 25000, Pakistan; nailaismail44@gmail.com; 4 Department of Pharmacy, Kohat University of Science and Technology, Kohat 26000, Pakistan; dr.mughalqayum@gmail.com; 5 Faculty of Medicine, Transilvania University of Brasov, 500019 Brasov, Romania; maria_elenacocuz@yahoo.com; 6 Faculty of Law, Transilvania University of Brasov, 500019 Brasov, Romania; catalina13.dinu@gmail.com; 7 Department of Medical Laboratory Technology, Faculty of Applied Medical Sciences, King Abdulaziz University, Rabigh 25732, Saudi Arabia

**Keywords:** cyclohexenone derivative, toxic effects, liver, kidney, pancreas, heart

## Abstract

Toxicity studies were conducted to provide safety data of potential drug candidates by determining lethal and toxic doses. This study was designed for pre-clinical evaluation of novel cyclohexenone derivative with respect to the acute and sub-acute toxicity along with the diabetogenic potential. Acute and sub-acute toxicity were assessed after intraperitoneal (i.p) injection of the investigational compound through selected doses for 21 days. This was followed by assessment of isolated body organs (liver, kidney, heart and pancreas) via biochemical indicators and histopathological techniques. No signs of toxicity were revealed in the study of acute toxicity. Similarly, a sub-acute toxicity study showed no significant difference in biochemical indicators on 11th and 21st days between treated and control groups. However, in blood urea nitrogen (BUN) and random blood glucose/sugar (RBS) values, significant differences were recorded. Histopathological evaluation of liver, kidney, pancreas and heart tissues revealed mild to severe changes in the form of steatosis, inflammation, fibrosis, necrosis and myofibrillary damages on 11th and 21st days of treatment. In conclusion, the median lethal dose of the tested compound was expected to be greater than 500 mg/kg. No significant change occurred in selected biomarkers, except BUN and RBS levels, but a histopathological study showed moderate toxic effect on liver, kidney, pancreas and heart tissues by the cyclohexenone derivative.

## 1. Introduction

**Figure 1:**
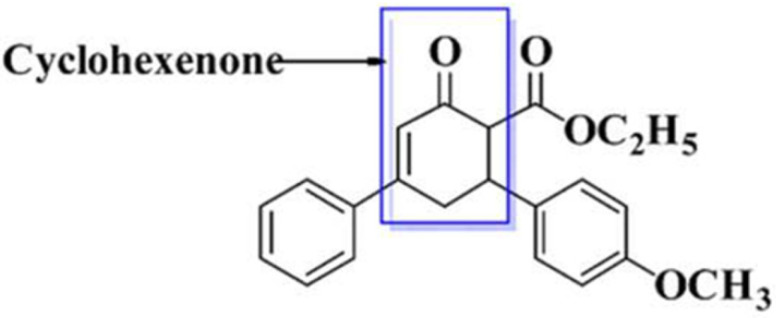
Ethyl 6-(4-methoxyphenyl)-2-oxo-4-phenylcyclohexe-3-ene-carboxylate.

In the process of drug discovery, pre-clinical testing of novel chemical entities involves assessment of both safety and efficacy. It is carried out in selected animal models through international standardized protocols. Broadly, it includes assessment of safety and efficacy through pharmacological studies with reference to the dose, frequency and route of administration. Promising outcomes at these steps can provide justified bases to proceed further into drug development [1]. Apart from pharmacological activity, toxicological assessment remained an integral part of the preclinical investigation. Toxicological evaluation is useful to provide data proving that the potential drug candidate is safe enough for next the phase of study i-e clinical testing. Such studies of toxicity testing have multiple utilities, such as an approximation of safe doses for humans and prediction of toxicity in susceptible organs. These provide scientific evidence regarding the safety profile, nature, variety and severity of adverse effects in association with its potential efficacy in usable doses, strength and concentration [2]. Cyclohexenones are cyclohexane derivatives that represent a carbonyl functional group and a double bond at positions C-1 and C-2, respectively. Their significant role is well known, including potential anti-inflammatory activity [3,4,5]. Furthermore, these compounds also have considerable antifungal, antibacterial, antiviral, anticancer, antiplasmodial and/or antimalarial activities [6,7,8,9,10,11,12]. Among these, one of the cyclohexenone derivatives is ethyl 6-(4-methoxyphenyl)-2-oxo-4-phenylcyclohexe-3-ene-carboxylate (Figure 1) [13].

Preliminary studies show that this derivative has a molecular formula of C_22_H_22_O_4_, melting point = 92–95 °C, relative front = 0.51 n-hexane/ethyl acetate (7:3). Infrared (KBr) spectrum shows υ_max_ cm^−1^: 3077 (Ar-H), 1689 (ketone C=O), 1735 (Ester C=O) and 2870 (Aliphatic C-H). ^1^H-NMR (CDCl_3_, 400 MHz) shows δ: 6.9–7.5 (m, Ar-H), 5.2 (s, ^1^H ethylene), 3.4 (s, ^3^H OCH3), 3.05 (d, ^2^H, *J* = 2.3), 2.9 (t, ^1^H *J* = 5.0, C-3) and 2.6–2.8 (q, 5H, CH_2_CH_3_, *J* = 7.0), and ^13^C-NMR (100 MHz, CDCL_3_) shows δ: 199.0 (C=O), 125–130 (Ar-CH), 112 (C-6), 40.2 (OCH_3_), 159.0 (C-19) and 44.39 (C-3). Similarly, EI-MS showed; m/z (rel. int.%) 351 (M+), CHN Anal. Calculated for: C, 75.41; H, 6.33; O, 18.26. Found: C, 74.81; H, 6.38. Furthermore, molecular docking studies revealed a strong affinity toward cyclooxygenase I and II (Cox-I and Cox-II) enzymes. It was observed in studies conducted in our laboratory that this derivative attenuated the pain aroused by vincristine in the rodent model, due to its possible antinociceptive and antioxidant effect [14]. To integrate its potential pharmacological activities with safety profile, toxicological studies are required to be conducted in an animal model.

Various studies have reported that Nonsteroidal anti-inflammatory drugs (NSAIDs) can induce renal toxicity, including acute renal failure [15,16,17]. Renal prostaglandin production is mediated mainly by activities of Cox-I and Cox-II enzymes. Since prostaglandins are not only produced during inflammation but also act as modulators of physiological functions i.e. when the volume of blood is compromised, prostaglandins play a role in the renal circulation (vasodilatation, renin secretion and Na^+^/H_2_O excretion). Hindering of prostaglandin formation by the consumption of NSAIDs in these situations can possibly lead to the development of a number of disorders of altered renal function [18,19]. In addition to the above adverse effects, liver toxicity has also been reported in the literature [20,21,22]. NSAIDs have evident toxicological potential regarding cardio-toxicities. This is mediated by Cox-II inhibition in the kidney, leading to fluid retention by decreased prostaglandins production. This results in a reduction of the glomerular filtration rate. Excess sodium and water retention lead to elevated blood pressure and thus develops cardiac complications. Increase in the production of reactive oxygen species in cardiac cells due to these drugs is also related to the initiation of damage to the myocardium [23,24,25]. Additionally, NSAIDs have possible membrane destabilizing effects on the pancreas, which may contribute to pancreatic toxicity [26,27,28]. Thus, this novel compound with affinity for Cox enzymes may be anticipated to disturb renal, hepatic, pancreatic and cardiac functioning.

For this reason, if the potential drug candidate is supposed to be used for anti-inflammatory and anti-nociceptive activities, its safety profile needs to be assessed via toxicological evaluation at selected body organs through histopathological and biochemical indicators. Therefore, this study is designed to evaluate the acute and subacute toxic effects of this compound on isolated body organs of the liver, kidney, heart and pancreas in an animal model through histological and biochemical indicating parameters.

## 2. Materials and Methods

### 2.1. Animals Breeding and Ethical Approval

Animals (mice) kept in a light and dark sequence (12 h each) at a suitable temperature (22 ± 2 °C) in cages, fed with water and laboratory standardized food, were raised in an animal house in the Department of Pharmacy, University of Peshawar (UoP). Specified age and weight range animals were selected for experimental studies. Ethical approval of preclinical studies on cyclohexenone derivative (CHD) was granted by the ethical committee of the Department of Pharmacy, UoP, via endorsement testament number 01/EC-18/PHARM; dated 16 October 2018. Experimental studies on mice were executed in congruence with UK Animals (Scientific Procedures) Act 1986.

### 2.2. Procedures

#### 2.2.1. Selection of Dose(s) and Route of Administration

The selection of dose(s) of this novel compound for its potential toxicity was based on previous studies by Jawad et al., conducted in the laboratory of the Department of Pharmacy, University of Peshawar, where he reported that this derivative had attenuated the pain aroused by vincristine in rodent model at doses of 30–45 mg/kg. The acute studies were performed on four groups constituting three treatment groups at doses of 250 mg/kg in group I, 350 mg/kg in group II and 500 mg/kg in group III, and a control group IV was used to understand the dose-dependency of the effect. Keeping in view the efficacy of doses, the maximum reported dose (45 mg/kg) of the said compound was taken as a starting dose for sub-acute toxicity study [14]. Similarly, Jawad et al. also provided information of LD_50_ value as beyond 240 mg/kg. To find the exact LD_50_ value, a higher dosage level of 250 mg/kg, 350 mg/kg and 500 mg/kg were selected to determine the possible lethal value of the dose [14].

Furthermore, the intraperitoneal (i.p) route of administration was selected, looking at the efficacy studies conducted by the same investigator. The researcher observed that this derivative had reduced the pain aroused by vincristine in an animal model using the same i.p route [14]. His finding was associated with the i.p route of administration; therefore, it was decided to correlate the toxicological effects with the pharmacological indication (analgesia) using the same route of administration. As its pharmacokinetic profile is not well established yet, and the researcher could not anticipate its physicochemical stability, site and rate of absorption in the gastrointestinal tract of the included animals; therefore, i.p route was considered to be more appropriate. It also provided better bioavailability with least exposure to unwanted physiological environment inside body.

#### 2.2.2. Acute Toxicity

A total of 24 mice (BALB/c), irrespective of their sex, having permissible weights of 20–30 gm, 8–12 weeks old, and kept in a light and dark sequence (12 h each) at an appropriate temperature (22 ± 2 °C), were used in the acute toxicity experiment. Toxicity studies were performed on both genders of the animals under study, aiming to assess the toxic effects in both sexes and to minimize the chances of inter-gender variation related to the said study. Keeping in view these facts, mice were selected irrespective of their gender [29,30]. Animals were equally divided into 4 groups i–e (*n* = 6); the groups treated with the compound were (groups I, II and III) and the control-normal saline treated group (group IV). Acute toxicity was assessed after intraperitoneal (i.p) injection of compound at the doses of 250 mg/kg to group I, 350 mg/kg to group II, 500 mg/kg to group III and saline to group IV. The adverse effects of the novel compound were noted at 30 to 60 min, then at 24, 48 and 72 h intervals and daily there-after, for a period of 14 days [30,31].

#### 2.2.3. Sub-Acute Toxicity

A total of 24 mice (BALB/c) irrespective of their sex, having acceptable body weights (20–30 gm), 8–12 weeks old and kept in a light and dark sequence (12 h each) at a suitable temperature (22 ± 2 °C), were tested in the sub-acute toxicity experiment. As stated earlier, the toxicity studies were performed on both the genders of the used animal, aiming to assess the toxic effects on both the sexes and to minimize the chances of inter-gender variation related to the said study [29,30]. Animals were categorized into group I (compound treated, *n* = 12) and group II (control/normal-saline treated, *n* = 12) [31,32,33]. Animal’s groups were treated in such a way that group I received an intraperitoneal (i.p) injection of the compound at a dose of 45 mg/kg/day for 21 days and group II received the vehicle and served as a control group.

#### 2.2.4. Pre-Clinical Observations and Survival

Signs of pre-clinical toxicity and mortality were noted. Pre-clinical observations included alteration in body weights, gait, posture and environmental interactions (cage–mates interactions and building nest). These observations were conducted weekly throughout the course of the acute as well as sub-acute study [34,35].

#### 2.2.5. Biochemical Assessment

During the course of treatment, blood samples were collected on 11th day (6 animals) and on 21st day (6 animals) from each group (sub-acute toxicity study groups). After centrifugation at 3000 rpm for 10–15 min, the separated serum was kept at 4 °C until analysis. The biochemical indicators evaluated were comprised of analysis of serum glutamic pyruvic transaminase (SGPT, also known as ALT) and serum glutamic oxaloacetic transaminase (SGOT, also known as AST) by utilizing analytical kits (ALT, AST; GO F400CH, Chema Diagnostica, Monsano, Italy); serum creatinine by using CR 0500CH, Chema Diagnostica, Monsano, Italy; serum blood urea nitrogen (BUN) using blood urea kits (Zone Industrille 61800 SEES, Normandy, France); random blood glucose level test using Abbott Glucometer; amylase tests through analytical kit by Randox Laboratories, London, UK; and troponin I and creatinine kinase-myocardial band (CK-MB) by utilizing standardized instrumental analytical technique through Finecare^TM^ Florescence Immunoassay analyzer. Data obtained from these tests were used to correlate with nature and level of toxicities in the hepatic, kidney, pancreatic and cardiac tissues [36].

#### 2.2.6. Histological Evaluation

For histological evaluation, on 11th and 21st days, 6 animals (on respective duration) were euthanized and their selected body organs (kidney, liver, heart and pancreas) were isolated for assessment of subacute toxicity. The selected organs/tissues being isolated were placed in 10% Formalin solution (neutrally buffered) for 6 to 48 hours. After fixation, they were cut into pieces of a proper thickness (5 µm) and placed in paraffin to form blocks. Different size sections were made through microtome and stained using H and E staining technique [37]. After staining, slides were examined using camera equipped light microscope (LABOMED LX400, iVu 3100, Auburn Court Fremont, CA, USA). The images acquired were assessed for any alterations. Those changes included hyperemia, necrosis, inflammatory cell aggregation, steatosis, fibrosis, interstitial edema, hemorrhage, degeneration of myocytes, glomerular injury and ectasia/tubular injury. These were subjectively assessed for scoring by a pathologist as none, minimum, mild, moderate and severe [38,39,40].

### 2.3. Statistical Analysis

Descriptive statistics and suitable t-test were performed using SPSS Version 22.0 (IBM, Armonk, NY, USA) and Graph-Pad Prism Software Version 5.01 (Graph-Pad Software Inc., San Diego, CA, USA). Effects during acute toxicity were assessed by applying one-way ANOVA (followed by Bonferroni multiple comparison). For parametric tests, the assumption of normality was calculated using the Skewness and Kurtosis analysis, followed by determination of z-values. Similarly, for homogeneity of variance, Levene’s test was applied (see Table 1 and Table 2). *p*-values less than 0.05 were considered as statistically significant.

## 3. Results

### 3.1. Acute Toxicity

Acute toxicity was evaluated using separated mice in groups, namely, group I (250 mg/kg), group II (350 mg/kg), group III (500 mg/kg) and group IV (control). After intraperitoneal injection (i.p) of selected doses of the cyclohexenone derivative (250 mg/kg, 350 mg/kg, 500 mg/kg) to respective groups, no signs of pre-clinical toxicity and mortality/death were observed in any treated group of mice up to a dose of 500 mg/kg. Therefore, lethal dose is expected to be greater than 500 mg/kg. Applying one-way ANOVA (followed by Bonferroni multiple comparison) revealed no significant change in the body weights of the group treated with compound as compared to the control group.

### 3.2. Sub-Acute Toxicity

#### 3.2.1. Body Weights

The mean values of body weights of mice at baseline, 7th and 11th day of treatment were 26.67, 27.17 and 26.83 g for control group and 26.5, 26.67 and 26.17 g for treated group, respectively. Applying independent sample t-test revealed no significant difference (*p* > 0.05) in body weights of treated and control groups.

The mean values of body weights at baseline, 1st, 2nd and 3rd week of treatment were 26.33, 26.83, 27.33 and 27.5 g for control group and 25.67, 26, 26.17 and 26.17 g for treated group, respectively. Applying independent sample t-test revealed no significant difference (*p* > 0.05) in body weights of treated and control groups.

#### 3.2.2. Biochemical Assessment

On 11th and 21st days, blood samples from treated and control groups were analyzed for alanine aminotransferase (ALT), aspartate aminotransferase (AST), serum creatinine, blood urea nitrogen (BUN), random blood glucose, serum amylase, cardiac troponin-I (cTn-I) and creatinine kinase-myocardial band (CK-MB). Results of these indicators are mentioned in the following headings and tables.

##### Liver

On 11th day of treatment, the mean value for ALT was 94.5 U/L in control and 98 U/L in treated group. Similarly, mean value for AST was 24 U/L in control and 25.5 U/L in treated group. By application of independent sample t-test, the *p*-value for ALT was 0.205 and for AST the *p*-value was 0.447. Thus, showing no significant difference of biochemical indicators values (ALT and AST) between treated and control groups on 11th day of treatment.

On 21st day of treatment, the mean value for ALT was 92.17 U/L in control and 94.5 U/L in treated group. Similarly, mean value for AST was 24.67 U/L in control and 24.5 U/L in treated group. Sample t-test revealed the *p*-value for ALT was 0.738, and for AST the *p*-value was 0.933, thus reflecting no significant difference in terms of biochemical indicators values between the treated and control groups on 21st day of treatment. All these values are depicted in Table 3.

##### Kidney

On 11th day of treatment, the mean value for BUN was 13.33 mg/dL in control and 26.67 mg/dL in treated group. Similarly mean value for serum creatinine was 0.517 mg/dL in control and 0.483 mg/dL in treated group. When independent sample t-test was aplied, *p*-values for BUN and serum creatinine were 0.00 and 0.461, respectively, thus revealing no significant difference in case of serum creatinine values but a significant difference occurs in BUN values between treated and control groups.

On 21st day of treatment, the mean value for BUN was 15.17 mg/dL in control and 18.5 mg/dL in treated group. Similarly, mean value for serum creatinine was 0.483 mg/dL in control and 0.5 mg/dL in treated group. By the application of independent sample t-test, significant difference was observed in values of BUN between control and treated group (*p*-value 0.03), but no significant difference occurred in values of serum creatinine between treated and control groups (*p*-value 0.734).

When biochemical indicators of treated group on 11th day were compared with treated group on 21st day, no significant difference was recorded between the said results of treated group (ALT *p*-value = 0.606, AST *p*-value = 0.629 and serum creatinine *p*-value = 0.734). However, in BUN values, a significant difference was observed between treated groups on 11th and 21st days (see Table 4).

##### Pancreas

On 11th day, half of the animals in control group were assessed for their amylase level, presenting mean values of 2683.17 U/L (in the treated group it was 2680.00 U/L). The same group of animals, when assessed for random blood glucose levels, showed means values of 83.83 mg/dL in control group and 47.83 mg/dL in treated group. Independent sample t-test showed a *p*-value of 0.831 for amylase level, showing no significant difference, but the random blood glucose has shown a *p*-value less than 0.05, thus showing a significant difference between the control and treated groups.

On 21st day, blood sample of the animals in the control group showed mean value of 2680.00 U/L; when evaluated for amylase level and in the treated group, it was 2672.00 U/L. The mean value of random blood glucose obtained in control group animals was 69.33 mg/dL, and in treated group of animals, the value was 56.67 mg/dL. The *p*–value obtained after sample t-test for amylase level was 0.515, thus showing no significant difference. The *p*-value obtained for random blood sugar level was 0.018, which is less than 0.05, thus showing a significant difference between control and treated groups. All these results are shown in Table 5 and Table 6.

##### Heart

On 11th day, half of the animals in control group, when assessed for their cardiac troponin-I levels, showed mean values of 0.29667 ng/dL and in the treated group 0.3 mg/dL (Table 7). Same animals when assessed for their CK-MB levels; the mean values of 11 U/L in control group and 13.83 U/L in treated group were recorded. Independent sample t-test showed *p*-value of 0.916 for cTn-I, thus, showing no significant difference and for CK-MB it was 0.672, also showing no significant difference between the control and treated groups on 11th day.

On 21st day, blood sample was assessed for cardiac troponin-I in control group of animals, for which the mean value of 0.2583 mg/dL and in treated group, mean value of 0.26167 ng/dL were recorded. The mean value of CK-MB obtained in control group animals was 13.83 U/L, and in treated group of animals, the value was 13.67 U/L. The *p*-value obtained after the sample t-test for cTn-I was 0.834, thus showing no significant difference. The *p*-value obtained for CK-MB level was 0.896, which is greater than 0.05, thus showing no significant difference between control and treatment groups.

When treated groups of animals were compared for their biochemical indicators on 11th and 21st days, no significant difference was observed. The *p*-value for amylase was 0.184, for random blood glucose level was 0.508, for cTn-I was 0.497 and for CK-MB was 0.786. However, random blood glucose level was significantly decreased by the end of 11th and 21st day of treatment, which presents no significant difference in the random blood glucose lowering effect in both treated groups of animals on 11th and 21st days (all these outcomes are shown in Table 7).

#### 3.2.3. Histopathological Evaluation

##### Effect on Liver

On 11th day, histopathological assessment of liver tissues revealed mild steatosis in all treated group animals. Mild hyperemia was observed in two animals, while mild fibrosis and inflammatory cell aggregation were observed only in one animal. No such pathological changes were observed in control group animals. Similarly, histopathological evaluation of liver tissues on 21st day revealed mild inflammatory cell aggregation in all treated group animals. Mild fibrosis and necrosis were observed in three and one treated group animal, respectively. Steatosis was observed in all animals ranging from mild to severe type. No such changes were observed in control group animals.

Images of liver tissue of mice on 11th day of treatment with normal saline have shown no signs of inflammation, hyperemia, steatosis and necrosis. However, images of liver tissue of mice on 11th day of treatment with cyclohexenone derivative (CHD) have shown some histopathological changes. Steatosis in hepatocytes was observed. Hyperemia and necrosis were also seen.

**Figure 2:**
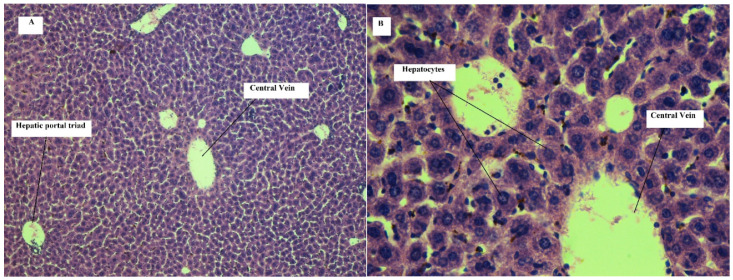

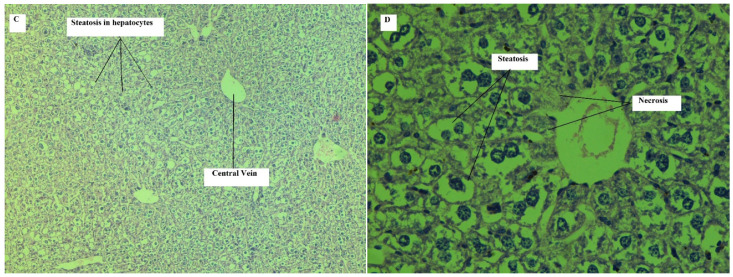
Liver of mouse treated with normal saline (H and E, 5µm, **A**: 100×, **B**: 400×): representative images of the liver of mouse treated with normal saline (i.p) for 21 days showing normal central vein, hepatocytes and hepatic portal triad. Liver of mouse treated with CHD (H and E, 5 µm, **C**: 100×, **D**: 400×): representative image of the liver of mouse treated with CHD (45 mg/kg/day, i.p) for 21 days. Steatosis and necrosis can be seen.

Figure 2A,B shows representative images of liver tissue of mice on 21st day of treatment with normal saline. Normal liver tissue architecture can be seen in these figures. Normal hepatocytes were observed. Central vein and hepatic portal triad can be seen. No inflammation, hyperemia, steatosis and necrosis were observed.

In Figure 2C,D, representative images of liver tissue of mice on 21st day of treatment with cyclohexenone derivative (CHD) are shown. Architecture changes were observed. Steatosis and necrosis in hepatocytes can also be seen. Those induced changes, present on 11th day, became more severe on 21st day of treatment. Further interpretation of the slides/images can be found in Table 8.

##### Effect on Kidney

On 11th day, histopathological assessment of kidney tissues revealed mild glomerular and tubular injury in all animals of treated group. In three animals, mild steatosis was observed. Mild hyperemia and inflammatory cell aggregation were detected only in one animal. These effects were observed in the animals of control group. Similarly, on 21st day, histopathological assessment has shown mild to moderate glomerular and tubular injury in treated group animals. Mild steatosis observed in five animals. In two animals of the treated group, mild fibrosis and necrosis were observed. Mild to moderate inflammatory cell aggregation was also seen in 4 animals of the treated group. Hyperemia was observed in one animal of the treated group. However, the animals of the control group did not show such pathological changes when assessed on the same day.

Kidney tissue of mice on 11th day of treatment with normal saline has shown normal renal tissue architecture, glomeruli, Bowman’s capsules and convoluted tubules. It was observed that glomeruli were normal and with intact structure. Similarly, it was found that normal Bowman’s capsule with endothelium was intact. No signs of inflammation, fibrosis, hyperemia and tubular injury were seen, but on 11th day of treatment with cyclohexenone derivative (CHD), the images of kidney tissue show multiple injured glomeruli with reduced size, inflammatory cells infiltration and increased blood flow (hyperemia) with tubular injury.

**Figure 3:**
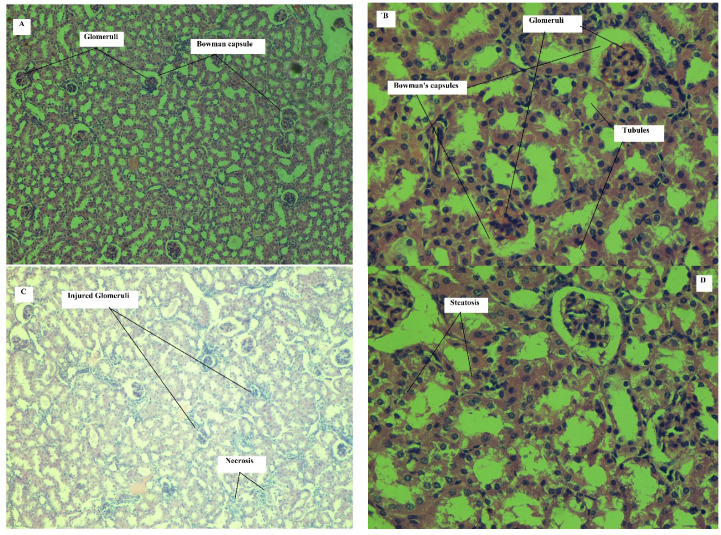
Kidney of mouse treated with normal saline (H and E, 5 µm, **A**: 100×, **B**: 400×): representative images of the kidney of mouse treated with normal saline (i.p) for 21 days showing glomeruli and Bowman’s capsules. Kidney of mouse treated with CHD (H and E, 5 µm, **C**: 100×, **D**: 400×): Representative images of the kidney of mouse treated with CHD (45 mg/kg/day, i.p) for 21 days showing injured glomeruli, steatosis and necrosis.

Figure 3A,B shows representative images of kidney tissue of mice on 21st day of treatment with normal saline. Normal renal tissue architecture can be seen in these images. Glomeruli, Bowman’s capsule and convoluted tubules are visible. Glomeruli were normal, and intact structure was observed. Similarly, Bowman’s capsules were normal and with intact endothelium. No inflammation, fibrosis, hyperemia and tubular injury were detected.

In Figure 3C,D, representative images of kidney tissue of mice on 21st day of treatment with cyclohexenone derivative (CHD) are shown. Multiple injured glomeruli with reduced size can be seen. Inflammatory cell infiltration and injured tubules are visible. Necrosis and steatosis can be seen in figures. The architectural changes presented on 11th day of treatment have shown an increase in frequency and severity on 21st day of treatment. Numerical scoring of the histopathological changes can be seen in Table 8.

##### Effect on Pancreas

Histopathological assessment on 11th day of treatment in mice has presented mild edema in their tissues in all animals of treated group. Necrosis was observed mildly in four animals. Mild inflammation was found in all animals except one, in which moderate inflammation was observed. Treated animals did not show hemorrhage and fat necrosis. These changes were not observed in control group animals. The results of assessment of pancreatic tissue on 21st day showed moderate edema in half of the animals in treated group and marked to severe edema in the remaining animals of the same group. There was mild necrosis in half of the group of treated animals and moderate in the remaining half with mild to moderate inflammation. No hemorrhage and fat necrosis were observed. In control group of animals, no such variations were observed on 21st day.

Histology of the pancreas has shown normal architecture in control group of animals after treatment with normal saline on 11th day of the treatment. Acinar cells were intact with endothelium with normal intra lobular ducts. No signs of edema, inflammation and necrosis were seen. However, histology of pancreas after treatment with cyclohexenone derivative (CHD) in treated group of animals, demonstrated alterations in normal histology on 11th day of treatment. Acinar cells were mildly necrotized in half of the animals. Mild edema and inflammation were observed in tissues surrounding acinar cells.

**Figure 4:**
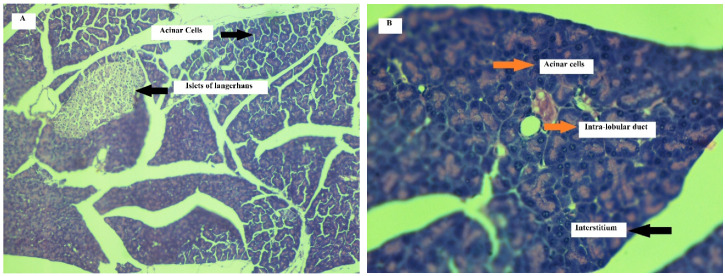

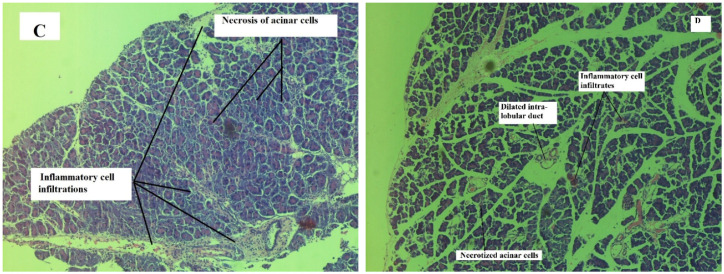
Normal saline treated pancreas of mouse (H and E stained, 5µm, **A**:100×, **B**: 400×): Representative image of pancreas of mouse treated with normal saline for 21 days showing normal intact acinar cells, islet of Langerhans with normal inter and intra lobular ducts. Pancreas of mouse treated with CHD (H and E stain, 5µm, **C**:100×, **D**: 400×): representative images of pancreas of mouse treated with CHD (45mg/kg/day, i.p) for 21 days showing moderately necrotized acinar cells with interstitial edema and inflammation.

Figure 4A,B represents histology of pancreas after treatment with normal saline in control group of animals on 21st day of treatment. The architecture of pancreas has presented normal histology. Acinar cells were intact with endothelium with normal intralobular ducts. No signs of edema, inflammation or necrosis can be seen in the figure.

Figure 4C,D signifies histology of pancreas after treatment with cyclohexenone derivative (CHD) in treated group of animals on 21st day of treatment. The architecture of pancreas has shown alteration in normal histology. Acinar cells were moderately necrotized in half of the animals of the treated group. Edema and inflammation was observed in tissues surrounding acinar cells. The alterations brought were increased in intensity on 21st day of treatment with CHD, as evident from the data shown in Table 9.

##### Effect on Heart

Histopathological assessment of heart in mice on 11th day of treatment with CHD revealed mild to moderate focal and multi focal damages. Minimum to mild inflammation was observed in half of the animals, but no myofibrillary damage and necrosis was observed. Any such changes were not observed in control group of animals. Assessment of heart tissue in treated group of animals showed moderate focal damages on 21st day. Multifocal damages were also observed in all animals. Moderate inflammation was found in the heart tissue of all animals. Mild myofibrillary damage was observed in half of the animals, except in one animal where moderate damage was found in myofibrils. No necrosis was found in treated group animals. No such detrimental changes were found in heart tissues of the control group animals on 21st day of treatment.

Histology of heart after treatment with normal saline in control group of animals on 11th day showed normal cardiac myocytes. Heart cells represented normal histology with mono or bi-nucleate muscle fiber. No signs of focal or multi focal damage were observed. No sign of inflammation and necrosis can be seen in the figure.

Heart cells in animals after treatment with cyclohexenone derivative (CHD) showed alterations in normal histology on 11th day of treatment. Cardiac myocytes also showed minimum focal damages. Multi-focal degenerations were observed with mild inflammation. However, no necrosis or myofibrillary damage was observed.

**Figure 5:**
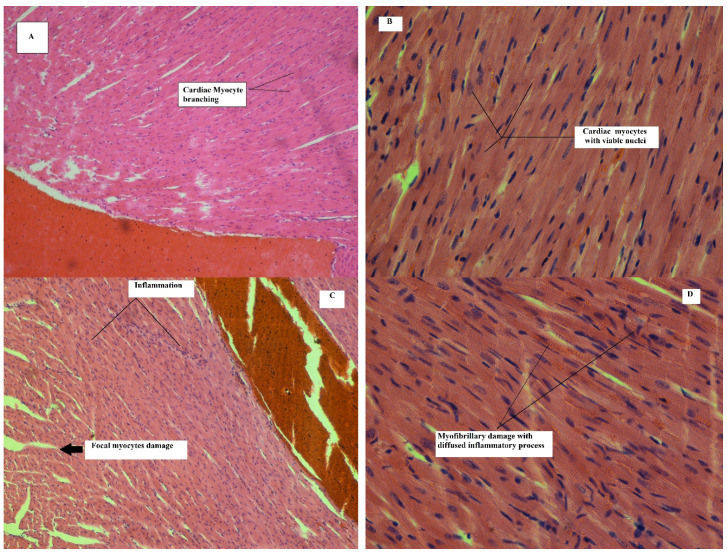
Normal saline treated heart tissue of mouse (H and E stained, 5 µm, **A**:100×, **B**: 400×): representative image ofheart tissue of mouse treated with normal saline for 21 days showing normal cardiac myocytes with mono or bi-nucleate muscle fiber. Heart of mouse treated with CHD (H and E stain, 5 µm, **C**:100×, **D**: 400×): representative images of heart tissue of mouse treated with CHD (45 mg/kg/day, i.p) for 21 days showing moderate focal myocytes damages with inflammatory cell infiltrations and myofibrillary damage with inflammatory process.

Figure 5A,B represents histology of the heart after treatment with normal saline in control group of animals on 21st day of treatment. The architecture of the heart presents normal cardiac myocytes. Heart cells represent normal histology with mono or bi-nucleate muscle fiber. No signs of focal or multi focal damage were observed. No sign of inflammation and necrosis can be seen in the figure.

Figure 5C,D represents histology of heart in animals, after treatment with cyclohexenone derivative (CHD) on 21st day of the treatment. Architecture of heart shows alterations in normal histology. Moderate focal damages can be seen in cardiac myocytes. Moderate multi-focal degenerations were observed. No necrosis and myofibrillary damage were observed. The alterations were increased in intensity on 21st day of treatment with CHD. Further details of the above-mentioned findings/outcomes are given in Table 10.

## 4. Discussion

The toxicological assessment of cyclohexenone derivative (CHD) for liver through biochemical indicators (ALT, AST) revealed no significant difference between treated and control groups. However, on 11th day of treatment, the mean values of corresponding indicators (ALT and AST) were higher in treated as compared to control group. On 21st day of treatment, the mean value of ALT was higher in treated group than the control group. However, no signs of any considerable difference in mean values of AST in control and treated group were found (approx. 25 for both treated and control groups).

In the light of the above, when liver injury occurs, the ALT leaks out into general circulation where its half-life is approx. 42 h. Thus, the value of this biochemical indicator was raised in blood but was not detectable beyond its half-life [41]. In the current study, the difference of mean values of ALT of control and treated group became minimal when the mean values of 11th and 21st day of treatment were compared. This could have been related to its short half-life, where it was released into general circulation due to damaged hepatocytes. Thus, the difference of mean values of ALT in control and treated group decreased on 21st day as compared to 11th day of treatment. Similarly, AST has shorter half-life than ALT. Thus, comparing the difference in mean values of control and treated group of AST indicated a decrease in difference on 11th and 21st days of treatment. The ALT and AST values of treated group were not significantly different from control group. Although histopathological changes in liver were observed, this may have been due to the hepatocytes. Cell membrane remained mostly intact, and significant damage was not observed. Consequently, the cytosolic enzymes (ALT, AST) were not leaking out to general circulation, and thus no significant difference were observed in biochemical tests used for liver function assessment. In the light of this, previous toxicity studies have also shown that the presence of histopathological changes does not correlate better with biochemical indicators [42,43], although in other studies histopathological and biochemical indicator changes occur concurrently. In such studies, the biochemical indicators increased significantly due the reason that notable hemorrhage and necrosis were observed in treated group. This led to the release of enzymes from hepatocytes cytoplasm, which caused a significant increase in these biochemical indicators in blood [44,45,46].

Although mean values of biochemical indicators (ALT and AST) were higher in treated group than the control group, the difference was statistically insignificant. With reference to the previous studies, the known hepatotoxic-drug (CCl_4_)-treated rodents showed mean values of ALT and AST to be more than 200 U/L and 30 U/L, respectively. This alteration by carbon-tetrachloride was majorly associated with liver injury through its metabolites that were generated during metabolism by cytochrome P_450_ [47,48,49,50]. In our current study, both on 11th and 21st days of treatment, the mean values of ALT were less than 100 U/L and the mean values of AST were less than 30 U/L. This shows that the said cyclohexenone derivative in a given dose and duration does not need to be very toxic to alter the ALT and AST values as compared to a known hepatotoxic drug. Drugs causing liver injury can be assessed through biochemical indicators, but their clinical significance is not clear. An increase of aminotransferase level > 3 upper limits of normal and jaundice (>3mg/dL) can be regarded as a risk of developing liver injury [51]. As in the current study, there was a slight increase in aminotransferase level, which may not indicate clinical significance.

Toxicological assessments of slides obtained from liver tissues revealed that mild steatosis occurred in treated animals on 11th day of treatment. Fibrosis and inflammatory cell aggregation occur in one treated animal, while hyperemia occurred in two treated animals. Such changes have shown increase in severity and frequency on 21st day of treatment, showing possible toxic effects of the administered compound. Biochemical indicators of liver have shown that the mean values of ALT in treated group were higher than control group. This increase may possibly be due to the fact that histopathological changes were observed in treated group animals, although ALT values of treated group were not significantly different from control group.

In connection to the above histological results of the liver, steatosis observed in liver tissue of mice in treated group could be related to β oxidation. Aspirin is a known cyclooxygenase inhibitor, and its induced steatosis in the liver is related to β-oxidation [52]. The molecular docking study of administered cyclohexenone derivative (CHD) is known to have affinity for cyclooxygenase enzyme that might be causing steatosis in liver tissue due to β-oxidation. Biochemical indicators of liver function (ALT and AST) on 11th day showed increase in mean values in treated group as compared to control group. This could be related to architectural damage that occurred on 11th day of treatment but without significant difference for the mentioned duration.

Toxicological assessment of cyclohexenone derivative for kidney through biochemical indicators (BUN and serum creatinine) revealed no significant difference in serum creatinine values of the treated and control groups. However, the BUN value has shown a significant difference between treated and control groups both on 11th and 21st days of treatment. In this connection, previous studies reported that in rodents treated with a known nephrotoxic agent, serum creatinine was more than 1mg/dL and BUN value more than 60 mg/dL. In those studies, in addition to increase in biochemical indicators values in treated group as compared to control group, histopathological evaluation revealed marked damage to kidney tissue. Architectural damage in those studies included necrosis, tubular injury, inflammation and vacuolization [47,53,54,55]. The results of current study showed that the mean values of serum creatinine, both on 11th and 21st days of treatment, were equal or less than 0.5 mg/dL. Similarly, the BUN mean values of treated group on 11th and 21st days were less than 60 mg/dL. This shows that the said cyclohexenone derivative (CHD) did not alter the biochemical indicators of kidney function as compared to known nephrotoxic agent.

Toxicological evaluation of cyclohexenone derivative through histopathological evaluation of slides from kidney tissues revealed that on 11th day of treatment, the glomerular and tubular injuries were mild in treated animals. These changes become moderate on 21st day of treatment, showing possible toxic effects of the administered compound, especially if used for extended period of time. Similarly, inflammation and fibrosis also appeared after 21st day of treatment. These effects support the increase in values of BUN in treated group as compared to control group. Some previous studies also demonstrated a significant increase in BUN values with histopathological changes in kidneys [46,56]. Similarly, with reference to the above, the decrease in size of glomeruli can be related to treatment with cyclohexenone derivative (CHD). Molecular docking studies indicated that this compound has affinity for cyclooxygenase enzymes. By blocking prostaglandins (PGs) synthesis, they block the vasodilation effect of PGs leading to vasoconstriction in the glomerulus. This ultimately resulted in reduced size of glomeruli and consequent damage [57]. Such histoarchitectural changes were also observed in our current study.

Toxicological assessment of cyclohexenone derivative for pancreas through biochemical indicators (amylase and random blood glucose) showed no significant changes in amylase level during treatment with CHD up to 21 days as compared to the control group. The amylase level when compared on 11th day and 21st day, between the control and the treated group of mice, indicated *p*-values that were not significant: 0.831 and 0.515, respectively. Treatment with CHD induced significant changes in random blood glucose levels in mice as compared to the control group animals on 11th and 21st days with *p*-values of less than 0.05 and 0.018 on respective days. The random glucose level was lowered during treatment up to 21 days in an animal model. The difference in the mean values of control group mice was minimal, but in the treatment group, the difference in the mean values at baseline, 11th and 21st day was greater, showing lower blood glucose level on 11th day and 21st day as compared to baseline. In treatment group, the glucose level was higher at the initial day of experiment and decreased up to the end of treatment. The random blood glucose values, when compared on 11th day of treatment and baseline, showed a *p* < 0.05, which is statistically significant. The *p*-value for random blood glucose level, when compared between the groups on the 21st day, gave a *p*-value that was statistically significant, i.e., 0.018. At baseline, the value was not significant with a *p*-value of 0.073.

With reference to the above, physiologically, glucose uptake by beta cells causes increase in ATP production, thus closing the ATP sensitive potassium levels. At the membrane potential of 50 mv, influx of calcium inside beta cells takes place and thus mediating the release of insulin. Studies found that NSAIDs like meclofenamic acid, which is also a Cox inhibitor, has been found to alter the blood glucose levels by altering the ATP sensitive potassium-channel. Its glucose lowering effect is brought by insulin release by inhibiting the ATP sensitive-potassium channels, which produces an increase in the potassium level inside beta cells of Langerhans and hence causes insulin release, triggering the hypoglycemic effect [26,58,59]. Acetylsalicylic acid and flufenamic acid also produce hypoglycemia due to increased insulin secretion and decreased gluconeogenesis by liver and reduced insulin clearance. In the present study, the glucose levels were not affected at baseline but showed marked hypoglycemia reaching at the end of sub-acute studies [26,60]. Opioids, such as tramadol and methadone, have also been found to produce hypoglycemia. It has been found that opioids are directly linked with mu-receptor agonism, resulting in direct uptake of glucose by hepatocyte and skeletal muscle cells. Some studies have found that the blood glucose lowering effect of tramadol and methadone was inhibited by naloxone. This revealed involvement of mu-receptors in the blood glucose lowering effect by opioids, with blood glucose levels monitored as low as 20 mg/dL [61,62,63]. In present case, toxicological investigation for cyclohexenone derivative showed that it has no effect on amylase activity but significantly lowered blood glucose levels up to hypoglycemic range in treated group of animals.

Histopathological assessment of pancreatic tissue slides revealed mild alterations on 11th day of treatment with cyclohexenone derivative. On 21st day, the histopathological parameters were intensified regarding induced changes. On 11th day, there was mild edema, necrosis and inflammation found in treated group of animals. No such changes were observed in control group of animals. These changes became moderate in intensity on 21st day, showing possible toxic effects due to treatment with CHD.

Furthermore, cyclohexenone derivative, being the inhibitor of cyclooxygenase enzyme, has been shown to increase hydrogen peroxide inside cellular compartments. This usually leads to cellular damage, releases cytokines and thus attracts inflammatory infiltrates due to degeneration of lipid, protein and nucleic acids. This may lead to cell apoptosis and necrosis and various pathological conditions [23]. The random blood glucose level in cyclohexenone-treated group showed a gradual decrease up to 21 days as compared to the baseline values, whereas the diabetogenic agents are required to raise the blood glucose levels beyond 300 mg/dL or 16.7 mmol/L. The blood tests for this agent in treated animals did not show random blood glucose levels above 300 mg/dL [64]. These results revealed that the test compound did not contain significant potential to induce diabetes for the said treatment.

The biochemical indicator of the heart (cTn-I and CK-MB) did not show any significant changes in the control and treated group by CHD for 21 days. When cTn-I level was compared between control and treated groups, no significant difference was observed with *p*-values of 0.916 and 0.834 on 11th and 21st days of treatment, respectively. Similarly, the value of CK-MB in control and treated group animals was found to be non-significant, with *p*-values of 0.672 and 0.896 on 11th and 21st days of dosing, respectively.

Nevertheless, cyclohexenone derivative, having affinity for cyclooxygenase, may induce cardio-toxicity by elevating the normal blood pressure. This effect is due to inhibition of Cox-II in kidney, which causes increased sodium and fluid retention and thus generates unwanted workload on heart due to elevated blood pressure, thus leading to elevated CK-MB and cardiac troponin-I levels due to any injury to the heart tissues [23]. Another mechanism causing increased levels of cTn-I and CK-MB is high level of reactive oxygen species in cardiac tissues. In normal physiological condition, reactive oxygen species are produced in mitochondria, which lead to synthesis of hydrogen peroxide via superoxide dismutase. This can be further converted to water molecules by glutathione peroxidase. NSAIDs have been shown to increase hydrogen peroxide species, which leads to cellular damage in myocytes due to degeneration of lipid, protein and nucleic acids. All these events lead to cell apoptosis and necrosis of the cardiac cells [23].

Histopathological assessment of heart tissues revealed that the level of cardiac troponin-I was less than 0.4 ng/mL, and for CK-MB blood test, the level was between 5–25 IU/L. The results showed that cyclohexenone derivative (CHD) did not induce any significant toxic effect on the biochemical indicators of the heart of mice as compared to toxicity producing agents, but it did alter the normal histology of the cardiac myocytes. As compared to control group, the treated group animals produced an alteration after CHD treatment. On 11th day, the heart tissue showed mild cardiac myocyte damage with inflammatory cell infiltrates, while on day 21, the heart tissue revealed mild damage with moderate adverse effects. Mild myofibrillary damage without necrosis was also observed. The damage to cardiac myocytes could be related to the affinity of the compound (CHD) to cyclooxygenase enzyme in kidneys, which, by inhibiting Cox-II enzyme, discontinues the vasodilation by prostaglandins (PGs). These processes result in sodium and water retention and elevate blood pressure, causing damage to heart tissues. Other possible mechanisms include increased production of hydrogen peroxide species, which can induce cellular damage to cardiac myocytes [23]. The histopathological alterations induced in tissues of heart suggested the possible toxic effects of this novel compound used for the treatment [65,66,67].

## 5. Conclusions

The acute toxicity studies of the novel compound have shown that the median lethal dose of the said compound is expected to be greater than 500 mg/kg, as no signs of toxicity and mortality were observed at this dose. Similarly, sub-acute toxicity revealed no significant changes in selected biomarkers except BUN and RBS levels. However, histopathological evaluation has shown mild to moderate toxic effects on liver, kidney, pancreas and heart tissues. These findings show that the novel compound may be toxic to selected body organs if used for sub-chronic or chronic duration.

## 6. Study Limitations and Future Plan

Findings of this study would have been more evident if the observed toxic effects had been correlated with the pharmacokinetic profile of the cyclohexenone derivative. Furthermore, it will be more beneficial to conduct toxicological studies of this novel compound on some additional vital body organs (like CNS and GIT) and for a longer duration (sub-chronic and/or chronic treatment) in an animal model. Therefore, we are planning to perform the assessment of pharmacokinetic parameters and to further extend the toxicological evaluation of the tested compound in an animal model.

## Figures and Tables

**Table 1:** Parametric tests, the assumption of normality by using the Skewness and Kurtosis analysis, followed by z-values.

Groups	Days of Treatment	Biochemical Tests	*n*	Mean	Skewness		z-Value	Kurtosis		z-Value
			Statistic	Statistic	Statistic	Std. Error		Statistic	Std. Error	
Control	11th day	Blood Urea Nitrogen (mg/dL)	6	13.33	0.248	0.845	0.293	−0.014	1.741	−0.008
		Serum creatinine (mg/dL)	6	0.517	−0.313	0.845	−0.370	−0.104	1.741	−0.059
		Alanine aminotransferase (ALT) U/L	6	94.50	−0.574	0.845	−0.679	−1.132	1.741	−0.650
		Aspartate aminotransferase (AST) U/L	6	24.00	0.433	0.845	0.512	−1.175	1.741	−0.674
		Valid N (listwise)	6							
	21st day	Blood Urea Nitrogen (mg/dL)	6	15.17	0.319	0.845	0.377	−1.171	1.741	−0.672
		Serum creatinine (mg/dL)	6	0.483	0.313	0.845	0.370	−0.104	1.741	−0.059
		Alanine aminotransferase (ALT) U/L	6	92.17	−0.036	0.845	−0.042	0.428	1.741	0.245
		Aspartate aminotransferase (AST) U/L	6	24.67	−0.224	0.845	−0.265	−1.864	1.741	−1.070
		Valid N (listwise)	6							
Treatment	11th day	Blood Urea Nitrogen (mg/dL)	6	26.67	0.435	0.845	0.514	0.586	1.741	0.336
		Serum creatinine (mg/dL)	6	0.483	0.313	0.845	0.370	−0.104	1.741	−0.059
		Alanine aminotransferase (ALT) U/L	6	98.00	−0.515	.845	−0.609	0.729	1.741	0.418
		Aspartate aminotransferase (AST) U/L	6	25.50	0.461	0.845	0.545	−1.260	1.741	−0.723
		Valid N (listwise)	6							
	21st day	Blood Urea Nitrogen (mg/dL)	6	18.50	0.000	0.845	0	−1.200	1.741	−0.689
		Serum creatinine (mg/dL)	6	0.500	0.000	0.845	0	−1.875	1.741	−1.076
		Alanine aminotransferase (ALT) U/L	6	94.50	0.401	0.845	0.474	1.635	1.741	0.939
		Aspartate aminotransferase (AST) U/L	6	24.50	0.255	0.845	0.301	−1.312	1.741	−0.753
		Valid N (listwise)	6							

**Table 2:** Parametric test for homogeneity of variance using Levene’s test.

	Levene’s Test for Equality of Variances—11th Day		Levene’s Test for Equality of Variances—21st Day	
	F	Sig.	F	Sig.
Blood urea Nitrogen (mg/dL)	0.741	0.410	1.250	0.290
Serum creatinine (mg/dL)	0.000	1.000	0.160	0.698
Alanine aminotransferase (ALT) U/L	0.877	0.371	2.094	0.178
Aspartate aminotransferase (AST) U/L	0.030	0.865	1.042	0.331

**Table 3:** Biochemical indicators: showing number of animals (*n*); mean ± standard deviation; and maximum, minimum and *p*-value of ALT and AST on 11th and 21st days of treatment.

Group	Statistical Parameter	Alanine Aminotransferase (ALT, 11th day)	Alanine Aminotransferase (ALT, 21st day)	Aspartate Aminotransferase (AST, 11th day)	Aspartate Aminotransferase (AST, 21st day)
Control (*n* = 6)	Mean ± SD	94.50 ± 5.089	92.17 ± 5.456	24.00 ± 3.464	24.67 ± 2.805
	Minimum	87	84	20	21
	Maximum	100	100	29	28
Treatment (*n* = 6)	Mean ± SD	98 ± 3.742	94.50 ± 15.681	25.50 ± 3.082	24.50 ± 3.834
	Minimum	92	72	22	20
	Maximum	103	120	30	30
Treated vs. control	*p*-value	0.205	0.738	0.447	0.933
Treated (11th day) vs. treated (21st day)	*p*-value	0.606		0.629	

**Table 4:** Biochemical indicators: showing number of animals (*n*); mean ± standard deviation; and maximum, minimum and *p*-value of BUN and creatinine of treated and control groups on 11th and 21st days of treatment.

Group	Statistical Parameter	Blood Urea Nitrogen (BUN, 11th Day)	Blood Urea Nitrogen (BUN, 21st Day)	Serum Creatinine (11th Day)	Serum Creatinine (21st Day)
Control	Mean ± SD	13.33 ± 1.75	18.50 ± 1.871	0.517 ± 0.075	0.500 ± 0.0894
	Minimum	11	16	0.4	0.4
	Maximum	16	21	0.6	0.6
Treatment	Mean ± SD	26.67 ± 2.73	15.17 ± 2.639	0.483 ± 0.075	0.483 ±0.0753
	Minimum	23	12	0.4	0.4
	Maximum	31	19	0.6	0.6
Treated vs. control	*p*-value	0	0.03	0.461	0.734
Treated (11th vs. 21st day)	*p*-value	0		0.734	

**Table 5:** Biochemical indicators: showing number of animals (*n*); mean ± standard deviation; maximum, minimum and p-value of serum amylase; and random blood glucose level on 11th and 21st days of treatment.

Group	Statistical Parameter	Serum Amylase (U/L) (11th Day)	Serum Amylase (U/L) (21st Day)	Random Blood Glucose (mg/dL) (11th Day)	Random Blood Glucose (mg/dL) (21st Day)
Control (*n* = 6)	Mean ± SD	2683.17 ± 26.649	2680 ± 23.421	83.83 ± 7.35	69.33 ± 9.07
	Minimum	2649	2655	73	60
	Maximum	2720	2700	90	85
Treatment (*n* = 6)	Mean ± SD	2680 ± 23.45	2672 ± 17.07	43.66 ± 13.89	56.67 ± 6.18
	Minimum	2655	2650	23	48
	Maximum	2710	2700	65	65
Treated vs. control	*p*-value	0.831	0.515	0.000	0.018
Treated (11th day) vs. treated (21st day)	*p*-value	0.184		0.508	

**Table 6:** Biochemical indicators: showing number of animals (*n*); mean ± standard deviation; and maximum, minimum and *p*-value of random blood glucose level weekly up to 21 days.

Group	Statistical Parameter	Random Blood Glucose (mg/dL)	Random Blood Glucose (mg/dL)	Random Blood Glucose (mg/dL)	Random Blood Glucose (mg/dL)
**Time Period**		**Baseline**	**7th Day**	**11th Day**	**-**
Control (*n* = 6)	Mean ± SD	83.57 ± 7.232	82.67 ± 4.179	83.83 ± 7.360	-
	Minimum	74	79	73	-
	Maximum	92	90	91	-
Treatment (*n* = 6)	Mean ± SD	82.17 ± 4.535	60.33 ± 3.011	47.83 ± 9.517	-
	Minimum	78	572	39	-
	Maximum	90	66	65	-
Treated vs. control	*p*-value	0.710	0.000	0.000	-
**Time Period**		**Baseline**	**7th Day**	**14th Day**	**21st Day**
Control (*n* = 6)	Mean ± SD	72.50 ± 4.722	75.50 ± 6.979	75.83 ± 6.555	69.33 ± 9.070
	Minimum	70	69	68	60
	Maximum	69	85	85	85
Treatment (*n* = 6)	Mean ± SD	80.83 ± 9.042	60.00 ± 7.563	56.17 ± 4.355	56.67 ± 6.186
	Minimum	74	79	73	48
	Maximum	92	90	91	65
Treated vs. control	*p*-value	0.073	0.004	0.000	0.018

**Table 7:** Biochemical indicators: showing number of animals (*n*); mean ± standard deviation; and maximum, minimum and *p*-value of cTn -I and CK-MB on 11th and 21st days of treatment.

Group	Statistical Parameter	Cardiac Troponin-I (ng/dL) (cTn-I, 11th Day)	Cardiac Troponin-I (ng/dL) (cTn-I, 21st Day)	Creatinine Kinase-Myocardial Band (U/L) (CK-MB, 11th Day)	Creatinine Kinase-Myocardial Band (U/L) (CK-MB, 21st Day)
Control (*n* = 6)	Mean ± SD	0.29667 ± 0.055	0.25833 ± 0.02483	11 ± 2.098	13.83 ± 1.871
	Minimum	0.25	0.23	9	8
	Maximum	0.4	0.27	14	12
Treatment (*n* = 6)	Mean ± SD	0.3 ± 0.0522	0.26167 ± 0.0286	13.83 ± 2.14	13.67 ± 2.160
	Minimum	0.26	0.22	12	11
	Maximum	0.4	0.3	16	17
Treated vs. control	*p*-value	0.916	0.834	0.672	0.896
Treated (11th day) vs. treated (21st day)	*p*-value	0.497		0.786	

**Table 8:** Histopathological evaluation scores of kidney and liver tissue slides of mice on 21st day of treatment.

Histopathological Findings	Animal Group-I						Animal Group-II					
	(Control)						(Treated)					
	1	2	3	4	5	6	1	2	3	4	5	6
Kidney												
Hyperemia	0	0	0	0	0	0	0	0	0	0	1	0
Necrosis	0	0	0	0	0	0	1	0	0	0	0	1
Inflammatory cell aggregation	0	0	0	0	0	0	1	0	0	1	2	1
Fibrosis	0	0	0	0	0	0	0	0	1	1	0	0
Glomerular injury	0	0	0	0	0	0	1	1	2	1	2	1
Steatosis	0	0	0	0	0	0	0	1	1	1	1	1
Ectasia/tubular injury	0	0	0	0	0	0	1	1	1	2	2	2
Liver												
Hyperemia	0	0	0	0	0	0	0	0	0	0	0	0
Necrosis	0	0	0	0	0	0	0	0	0	0	0	1
Inflammatory cell aggregation	0	0	0	0	0	0	1	1	1	1	1	1
Fibrosis	0	0	0	0	0	0	1	0	0	1	0	1
Steatosis	0	0	0	0	0	0	2	1	3	2	2	2

0 none; 1 mild; 2 moderate; and 3 severe.

**Table 9:** Histopathological scoring of pancreatic tissues of control and treated groups of mice during 11th and 21st days of treatment.

Scoring of Pancreas Tissue Slides												
**on 11th Day**	**Control Animals**						**Treated Animals**					
**Parameters**	**1**	**2**	**3**	**4**	**5**	**6**	**1**	**2**	**3**	**4**	**5**	**6**
Edema	0	0	0	0	0	0	1	1	1	1	1	1
Necrosis	0	0	0	0	0	0	1	1	1	0	1	0
Inflammation	0	0	0	0	0	0	1	1	2	1	1	1
Hemorrhage	0	0	0	0	0	0	0	0	0	0	0	0
Fat necrosis	0	0	0	0	0	0	0	0	0	0	0	0
**on 21st Day**	**Control Animals**						**Treated Animals**					
**Parameters**	**1**	**2**	**3**	**4**	**5**	**6**	**1**	**2**	**3**	**4**	**5**	**6**
Edema	0	0	0	0	0	0	2	2	3	3	2	4
Necrosis	0	0	0	0	0	0	2	1	2	1	1	2
Inflammation	0	0	0	0	0	0	1	1	2	2	1	3
Hemorrhage	0	0	0	0	0	0	0	0	0	0	0	0
Fat necrosis	0	0	0	0	0	0	0	0	0	0	0	0

0 none; 1 mild; 2 moderate; 3 marked; and 4 severe.

**Table 10:** Histopathological scoring of heart tissues of control and treated group of mice during 11th and 21st days of treatment.

Scoring of Heart Tissue Slides												
**on 11th Day**	**Control Animals**						**Treated Animals**					
**Parameters**	**1**	**2**	**3**	**4**	**5**	**6**	**1**	**2**	**3**	**4**	**5**	**6**
Focal myocytes damage	0	0	0	0	0	0	2	2	3	2	2	2
Multifocal degeneration	0	0	0	0	0	0	2	2	2	2	2	2
Multifocal degeneration with inflammatory process	0	0	0	0	0	0	0	2	2	0	1	1
Myofibrillar degeneration/diffuse inflammatory process	0	0	0	0	0	0	0	0	0	0	0	0
Necrosis with diffuse inflammatory process	0	0	0	0	0	0	0	0	0	0	0	0
**on 21st Day**	**Control Animals**						**Treated Animals**					
**Scoring Parameters**	**1**	**2**	**3**	**4**	**5**	**6**	**1**	**2**	**3**	**4**	**5**	**6**
Focal myocytes damage	0	0	0	0	0	0	3	3	3	3	3	3
Multifocal degeneration	0	0	0	0	0	0	3	3	3	3	3	3
Multifocal degeneration with inflammatory process	0	0	0	0	0	0	1	2	3	3	3	2
Myofibrillar degeneration/diffuse inflammatory process	0	0	0	0	0	0	0	0	2	3	2	2
Necrosis with diffuse inflammatory process	0	0	0	0	0	0	0	0	0	0	0	0

0 none; 1 minimum; 2 mild; 3 moderate; and 4 severe.

## Data Availability

Not applicable.
